# An Investigation of Influential Users in the Promotion and Marketing of Heated Tobacco Products on Instagram: A Social Network Analysis

**DOI:** 10.3390/ijerph19031686

**Published:** 2022-02-01

**Authors:** Jiayan Gu, Lorien C. Abroms, David A. Broniatowski, W. Douglas Evans

**Affiliations:** 1 Department of Prevention and Community Health, Milken Institute School of Public Health, The George Washington University, Washington, DC 20052, USA; lorien@gwu.edu; 2 Department of Engineering Management and Systems Engineering, The George Washington University, Washington, DC 20052, USA; broniatowski@gwu.edu

**Keywords:** social media, heated tobacco products, IQOS, Instagram, social network

## Abstract

While an increasing body of the literature has documented the exposure to emerging tobacco products including heated tobacco products (HTPs) on social media, few studies have investigated the various stakeholders involved in the generation of promotional tobacco content. This study constructed a social network of Instagram users who posted IQOS content, a leading HTP brand, between 1 January and 5 April 2021 and identified users who positioned near the center of the network. We identified 4526 unique Instagram users who had created 19,951 IQOS-related posts during the study period. Nearly half of the users (42.1%) were business accounts authorized by Instagram, among which 59.0% belonged to Personal Goods and General Merchandise Stores and 18.1% belonged to Creators and Celebrities. For users with higher in-degree, out-degree, betweenness, and closeness centrality in the network, the majority of them were accounts directly associated with IQOS (e.g., containing “iqos” in username) or related to tobacco business as self-identified in the bio. Our findings further refine the social media marketing presence of tobacco products and suggest that the current self-regulatory efforts led by social media platforms are far from enough.

## 1. Introduction

Heated tobacco products (HTPs), also known as heat-not-burn tobacco products, are one of the alternative nicotine delivery systems (ANDS) that have successfully entered the global tobacco market in recent years [1,2]. The global HTP market size was valued at USD 7.3 billion in 2019 and was estimated to expand at a compound annual growth rate of around 32.8% over the forecast period from 2020 to 2027 [3]. HTPs are a battery-powered, pen-like device that generates an inhaled nicotine aerosol by smoldering shredded leaf tobacco instead of combusting tobacco [4]. Tobacco manufacturers have been explicitly or implicitly using reduced risk claims to promote HTPs across many countries [5]. However, research not commissioned by tobacco manufacturers cautions that while HTPs may expose users to lower levels of some toxicants than cigarettes, they contain higher levels of other toxicants that may lead to increased risks of impaired vascular endothelial function, pulmonary effects, and liver toxicity [4,6,7,8,9]. In addition, nicotine addiction is a chronic and relapsing illness characterized by compulsive seeking and use, despite user awareness about risks and desire to quit [10]. These facts raise health concerns specifically for youth and young adults who are more vulnerable to nicotine addictiveness [11].

Inaccurate marketing claims, especially “no smoke” claims and “high-tech” design features, are suggested to decrease users’ assessments of potential harms associated with a new tobacco product and thus increase the possibility of tobacco use [12]. Among various exposures to pro-tobacco content, social media marketing tactics used by the tobacco industry are one of the most influential information sources. A content analysis of 112 leading brands of e-cigarettes, hookah, cigars, cigarettes, and smokeless tobacco products on six platforms (i.e., Instagram, Facebook, Twitter, YouTube, Pinterest, and Tumblr) suggested that most e-cigarettes, hookah, and cigar brands had official pages on at least two platforms and brand pages rarely used age gating, did not display warning messages, generally posted product images alone and used hashtags unrelated to tobacco [13]. Similar evidence on extensive exposures to ANDS across different social media platforms was also found in a number of studies [14,15,16,17,18,19,20] and a significant amount of such exposures featured content related to youth [19].

In 2019, following more than a year of an intense world-wide campaign led by The Campaign for Tobacco-Free Kids which urged leading social media platforms to take immediate actions on the promotion of ANDS on their platforms [21], Facebook and Instagram responded by announcing new policies to prohibit influencer marketing of tobacco products [22]. Although paid advertising of tobacco products had long been banned on both platforms, the new announcement asserted to extend existing policies to prohibit tobacco companies from promoting all kinds of tobacco products by approaching users with a large number of followers known as “influencers”. However, the enforcement and effectiveness of this new announcement remain questionable, especially considering the lack of definition of “influencers” and extensive user-generated content related to tobacco on the platforms. A study found that on Instagram, the promotion of IQOS was overwhelmingly from online retailers and fan communities, featuring IQOS with terms of fashion, health, flavor, and cessation [23]. Another study in Czech found celebrities and influencers actively presented IQOS as a gateway to an aspirational, healthy, attractive, and celebrity lifestyle in their posts and videos since 2018 on Instagram [24].

Investigating different stakeholders who play an influential role in the promotion of HTPs and other ANDS is important to help understand how the tobacco industry is trying to normalize the use of ANDS and inform regulations of tobacco marketing on social media. While most existing studies on the exposures to tobacco products on social media have focused on the presentation, characterization, or description of tobacco content [19,20,23,24,25], little attention has been paid to various stakeholders who are involved in the production of pro-tobacco content on social media. This study aimed to contribute to this field by applying social network analysis to identify and characterize the network among Instagram accounts who were involved in publishing posts related to IQOS, a leading HTP brand on Instagram. Specifically, our research questions included: (1) what were the structural characteristics of IQOS network on Instagram; (2) were certain Instagram accounts more important (e.g., central) than others in the dissemination of IQOS-related content within the network; (3) if certain accounts played more central roles in disseminating the concept of IQOS, what were the characteristics of these Instagram accounts?

## 2. Materials and Methods

Between May and July 2021, we collected a dataset of public Instagram posts that contained the hashtag #IQOS from 1 January 2021 and 5 April 2021 using Instaloader [26], a software that scraped Instagram for images and associated metadata given a specific hashtag. The hashtag search strategy has been used in previous research that examined public health content on Instagram [19,23,27]. Metadata returned by Instaloader includes each post’s media information (i.e., image, captions, and timestamp), the URL link, the number of likes and comments, and a user ID. Since the geolocation information was only available for a small proportion of posts, we labeled each post a primary language based on the caption and hashtag information using Langdetect, a language detection library in Python, as a proxy of its location [28].

Once posts that contained #IQOS during the study period were collected, each account who had published IQOS-related post(s) on Instagram during the observation time were considered as a member (i.e., node) within the network and composed a new database. Next, we collected the account profile data including username, bio information, following and followed relationships, business category, and whether it was a verified account using Instaloader. To further characterize the influential level of each account, we defined five categories based on their number of followers: normal (<1000 followers), nano (1000–10,000 followers), micro (10,000–50,000 followers), mid-tier (50,000–500,000 followers), and macro (>500,000 followers). The number of followers shows the potential reach of an account. On Instagram, an account can select to convert from a personal profile to a business account to access certain features that help better understand the engagement and track performance of the account. There are a number of business categories defined by Instagram, which is displayed at the top of the profile after converting to a business account [29].

In the social network representation, each node represented an account who had published at least one post with the hashtag #IQOS on Instagram during the study period. Each directed edge represented the following relationship between two nodes. For example, a directed link from A to B existed when A followed B on Instagram. The color of each node was given by whether it was a business account. The blue nodes referred to business accounts authorized by Instagram. In contrast, the green nodes referred to personal accounts. The size of each node represents the level of in-degree of that node [30]. A node with a higher in-degree meant it was followed by more nodes and the content generated by it would reach more nodes within the network. The visualization of the social network was done in Gephi, an open-source network analysis and visualization software package [31].

Following the network visualization, we calculated several network metrics to provide an overview of the network structure. We first examined the number of components and identified the largest component to understand how well connected the network was. Then we calculated the network density, average degree, and average clustering coefficient for both the whole network and the largest component. The density of a network refers to the total number of relational edges divided by the total possible number of relational edges, which ranges from 0 to 1 with the lower limit representing to a sparse network with no relationships and the upper limit representing a dense network with all possible relationships [30]. The average degree is simply the average number of edges per node in the graph. The average clustering coefficient can indicate the “small-world effect”, or an overall indication of the clustering in the network [30].

To identify nodes that played a more central role within the network, we measured four types of centralities of the nodes to assess whether they were near the center of the network. The in-degree centrality represented the number of edges coming into the node [30]. In this case, the content associated with IQOS published by nodes with higher in-degree centrality was more likely to be viewed by other nodes. The out-degree centrality measured the number of edges coming out of the node [30]. On Instagram, the law of reciprocity often motivates an account to follow others in exchange for a mutual follow [32]. Thus, the out-degree centrality to some extent represented how central a node was in terms of sociability or expansiveness. In addition, we also measured betweenness centrality, which was a measure of the potential for control as a node acting as a gatekeeper or a bridge controlling the flow of information, and closeness centrality, which provided information on the mean shortest path geodesic distance between the node and all other nodes reachable from it [30]. After the centralities were calculated, we conducted a descriptive analysis of the top five accounts under each centrality measure in terms of their characteristics and posts associated with IQOS. All posts and user profiles in this dataset were publicly available on Instagram and presented anonymously in this manuscript to protect privacy.

## 3. Results

### 3.1. User Characteristics

During the study period, we identified a total of 19,951 Instagram posts that contained a hashtag of #IQOS, published by 4526 unique Instagram accounts. Table 1 presents the descriptive characteristics of these accounts. The majority of accounts had only one IQOS post (72.5%) and were normal accounts with less than 1000 followers (74.9%). Although not so large in number, there were 126 micro accounts (2.8%) with followers between 10,000–50,000, 25 mid-tier accounts (0.6%) with followers between 50,000–100,000, and 2 macro accounts who had over 500k followers on Instagram. Almost half of the users were Instagram-authorized business accounts (42.1%), among which 59.0% belonged to the category of personal goods and general merchandise stores and 18.1% belonged to creators and celebrities. Only 0.1% were verified users, which referred to an account verified by Instagram as the authentic presence of the public figure, celebrity, or global brand it represents. The proportion of languages was roughly consistent with the popularity of IQOS in different countries, where almost one-third of the accounts were Japanese (29.2%), followed by English (19.6%), Italian (13.9%), and Russian (11.4%).

### 3.2. Social Network Analysis

**Figure 1:**
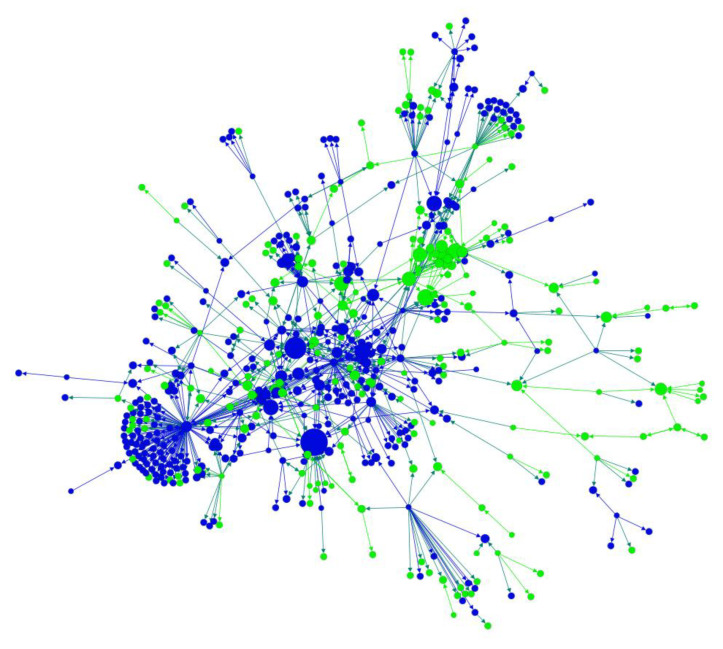
The largest component of the network of Instagram accounts with #IQOS post(s) between 1 January 2021 and 5 April 2021. (Each node represented an account who had post(s) with the hashtag #IQOS on Instagram during the study period. Each directed edge represented the following relationship between two nodes. Blue nodes referred to business accounts authorized by Instagram and green nodes referred to personal accounts. The size of each node represents the level of in-degree of that node).

Table 2 presents the network metrics for the whole network and the largest component, respectively. In total, there were 4526 nodes in the network, among which 82.1% (N = 3714) were isolated, meaning they were not following or followed by any other nodes in the network. The remaining network had 812 nodes, 1082 directed edges, and 98 connected components. The graph density for the whole network was 0.004, suggesting it was a quite loose-knit network. Each node had an average of 1.333 connections. The average clustering coefficient was 0.171, indicating that the network is a relatively small world. The largest component of the network contained 532 nodes and 829 directed edges (see Figure 1). It consisted of 65.5% of all non-isolated nodes and was denser than the whole network. The average degree of this cluster was 1.558 and the average clustering coefficient was 0.181. It is noted that 54.8% of the nodes were business accounts authorized by Instagram (i.e., blue nodes), which were heavily entangled with personal accounts (i.e., green nodes). There were several apparently larger blue nodes within the network, suggesting that these business accounts had a higher level of in-degree and were followed by more accounts involved in the promotion of IQOS.

To investigate nodes that were positioned more centrally within the network, we identified the top five nodes for each of the four common centrality measures (i.e., in-degree, out-degree, betweenness, and closeness) (Table 3). We found that for nodes with higher in-degree centrality, two of them were accounts with over 10k followers. Four out of five were business accounts and three of them contained characters of “iqos” in the username, suggesting a direct association with the brand IQOS. For nodes with higher level of sociability and expansiveness (i.e., out-degree centrality), three out of five were business accounts and two of them were IQOS-associated. As an indicator of a node’s centrality in terms of the degree to which the node mediated in information or controlled the information paths (i.e., betweenness centrality), three out of five were IQOS-associated business accounts while one non-business account had over 140k followers. Similarly, four out of five nodes with the highest closeness centrality were business accounts containing “iqos” in their username.

### 3.3. Descriptive Analysis

To better understand the characteristics of these central nodes with higher centrality in the network, we conducted a descriptive analysis of these accounts and the IQOS posts they generated. Table 4 presents the characteristics of these accounts by category with example posts. Excluding duplicated accounts, there were a total of 13 central accounts included for analysis. Among them, six accounts contained “iqos” in the username and were assumed to be directly associated with the brand IQOS. These accounts included IQOS Instagram account representing a specific region or country, local IQOS retailer/reseller stores, and IQOS case/skin personalization service providers. Most of these accounts’ posts showed the product of IQOS in the center and a link directing to an external source where viewers could get access to the selling or service of IQOS. Another category of accounts (*n* = 5), though not directly associated with IQOS, were tobacco related as suggested by their username or profile bio. For example, an account may include “vape” or “hnb” in the username or indicate that it is a tobacco product retailer in the bio. These accounts did not exclusively focus on IQOS but also covered other tobacco products. The posts from these accounts included promotional content of a wide range of tobacco products but the format was quite similar to the previous category. The remaining category was celebrity or social media influencers rather than specifically tobacco-focused accounts. The IQOS posts created by them were usually a daily usage scenario of the product. The number of posts with #IQOS in total was only one for each account, but the number of followers they could reach was noticeably large.

In summary, we found that the overall network was relatively sparse with a large proportion of isolated nodes. However, we identified a large component within the network that was overwhelmingly connected by business accounts. By identifying nodes that positioned near the center of the network, we found that the majority of them were either directly associated with the brand IQOS or related to tobacco business. The posts created by these accounts normally featured content of the product and provided external links directing viewers to access tobacco products or services.

## 4. Discussion

This study extends previous research and offers key insights into the users who are involved in the generation of IQOS content on Instagram, a social media platform popular among youth and young adults. We structured and characterized a social network of users who posted IQOS content on Instagram during the study period and identified central nodes based on several network centrality measures. While the network of IQOS was relatively loose in general, a large component was identified in the network with overwhelmingly business accounts. Unlike individual users, business accounts usually use Instagram to reach and grow new customers and engage with an existing audience. They are also motivated to follow other business accounts as a means of reciprocity and growing the size of followers. A large cluster of accounts who created IQOS content was found being connected with each other on Instagram suggested a growing community consisting of worldwide marketing efforts of IQOS on social media.

Our findings further refine the social media marketing presence of IQOS, which aligns with prior research that has documented and characterized emerging tobacco products across different social media platforms [15,16,17,18,19,20,23,24,25,33]. We found that promotional IQOS posts were largely driven by stakeholders along the IQOS business chain. On Instagram, there are over 1500 categories that a business can choose from, and the category can inform Instagram’s search algorithm to display the business when people search for it [34,35]. In our sample, around half of the total users were business accounts, among which nearly 80% of them were identified as Personal Goods and General Merchandise Stores and Creators and Celebrities on Instagram. This suggests that the creators of IQOS content on Instagram were mostly online retailers of IQOS or paid social media influencers who presented the use of the product for marketing. This finding is consistent with a previous study that found in a sample of Instagram posts on HTPs, online retailers represented about 60% and HTP users represented 35% of the sample [23]. This also conforms to previous characterizations of IQOS on social media that the majority of the content were either dedicated to the marketing of IQOS or crafted by individuals who had some experience with using the product [20]. Our findings also suggested that the majority of the IQOS posts were from users with less than 1k followers (74.9%). Celebrities or influencers who had over 50k followers only accounted for 0.6% of the dataset. While this is a promising finding that fewer celebrities or influencers are involved in the promotion of tobacco products, a close monitoring of such accounts is important as they usually have a larger reach of tobacco-naïve people.

Understanding the characteristics of central nodes within the network can help reveal the key stakeholders who have been actively promoting IQOS on social media and those who may play a more important role in controlling the normalized information of a newly introduced tobacco product. Interestingly, we found the majority of nodes which were more central within the network were IQOS associated accounts, including IQOS brand accounts presenting a specific country or region, online IQOS retailers, and IQOS customization service providers. These accounts usually dedicatedly create IQOS content that highly aligns with the official product positioning set by the manufacturer, which features high-tech, fashion-conscious, and personalized design. Currently, tobacco brand associated accounts and online tobacco retailers are not regulated or prohibited by Instagram. They can even convert to a business account that enables more performance tracking tools and create promotional content without any age restrictions on the platform. 

The data from our study also indicate the limitations of relying on self-regulation by platforms on the social media marketing of tobacco products. The 2019 campaign led by The Campaign for Tobacco-Free Kids pointed out that while leading social media platforms had long banned paid advertising for tobacco products, pro-tobacco content generated by influencers created a loophole allowing rampant marketing of tobacco products [21]. In response, some platforms, including Facebook and Instagram, extended their policy to prohibit the use of paid influencers to promote tobacco products in the face of pressure from the public health community [22]. However, the policies are obviously hard to enforce without a clear definition of influencers and how platforms are able to identify paid advertising activities between tobacco companies and social media influencers. Our findings also suggest that in addition to influencers, brand-associated accounts and a substantial number of online retailers are worryingly neglected by current regulations. These accounts not only make up the majority of users who created the product content but are also positioned near the center of the network. The content created by these accounts is likely to be more influential in deciding how people perceive a tobacco product. Therefore, it is urgently needed to amend the branded content policy or advertising policy to regulate marketing efforts by various stakeholders including influencers, retailers, and brand-associated accounts. Previous experience indicated that external regulatory efforts by governments can also put pressure on social media companies by requiring transparency on certain topics. At the same time, we should note the difficulties in regulating user-generated content on social media and consider other interventional approaches such as counter-marketing campaigns targeting vulnerable populations. 

This study has several limitations. First, we used a hashtag search strategy to identify and collect IQOS-associated posts on Instagram. Although this approach has been commonly used in previous research that involved collecting public Instagram data [19,23,27] and we also tried multiple hashtags related to IQOS to avoid missing any representative hashtags in data collection, it undeniably would miss IQOS-associated posts that do not contain any product-related hashtag, especially those IQOS accounts that refrained from using the hashtag perhaps as a way of evading regulation and academic scrutiny. Consequentially, the hashtag search strategy would underestimate the network of accounts involved in the promotion of IQOS. However, a previous study on e-cigarettes has suggested that most posts on Instagram had a hashtag to the product [13]. We believe the hashtag search strategy may introduce a few, but not significant, biases into data collection. Second, Instagram in 2018 launched a geofencing feature which allowed business accounts to post their content in selected countries instead of being universally available [36]. The study was conducted in the United States, so it did not include posts and accounts that were geofenced (e.g., @iqos_jp, @iqos_co, @iqos_id). It is therefore noted that the study may under-reflect the presence of users who are influential in specific regions excluding the United States such as the geofenced IQOS official accounts in Japan, Colombia, and Romania. Third, the study focused on IQOS posts on Instagram during a specific time period from January to early April 2021. Our findings may not generalize to other time periods. However, the purpose of this study was not to provide a universal description of the landscape of IQOS promotion on Instagram but to offer a cross-sectional investigation where IQOS was rapidly growing around the world. Though the results may underestimate the actual network and under-reflect influential users in the promotion of IQOS, we believe our sample size and analyses were sufficient for our study purpose as well as for reaching the conclusions.

Results from our analysis may provide guidance for future research in the following ways. Future social media analysis of IQOS and other ANDS should note the restricted features of different platforms such as geofencing and accordingly develop a data collection approach as comprehensive as possible. Future research may also extend our findings by systematically analyzing the content created by business accounts and investigating what content is more likely to be endorsed by social media users.

## 5. Conclusions

As a growing ANDS with design features appealing to youth and young adults, a close monitoring of social media marketing of IQOS and other HTPs is critical to avoid increasing tobacco use among a new generation. Our results suggest that the current self-regulatory efforts led by social media platforms are not sufficient. More refined and feasible regulatory action, especially limiting the marketing efforts by official accounts, online retailers, and celebrities, is needed to restrict the proliferation of promotional content associated with tobacco products.

## Figures and Tables

**Table 1:** Characteristics of Instagram accounts with IQOS posts (N = 4526).

Account Characteristics	N (%)
Number of IQOS posts	
1 post	3280 (72.5)
2–4 posts	723 (16.0)
5–9 posts	207 (4.6)
10–50 posts	278 (6.1)
50–100 posts	27 (0.6)
>100 posts	11 (0.2)
Number of followers ^a^	
Normal	3390 (74.9)
Nano	983 (21.7)
Micro	126 (2.8)
Mid-tier	25 (0.6)
Macro	2 (0.0)
Business account ^b^	
Yes	1903 (42.1)
No	2623 (58.0)
Business category	
Personal Goods and General Merchandise Stores	1110 (59.0)
Creators and Celebrities	341 (18.1)
Home Services	92 (4.9)
Restaurants	64 (3.4)
General Interest	44 (2.3)
Others ^c^	232 (12.2)
Verified account ^d^	
Yes	6 (0.1)
No	4520 (99.9)
Primary language ^e^	
Japanese	1322 (29.2)
English	888 (19.6)
Italian	630 (13.9)
Russian	518 (11.4)
Others	1168 (25.8)

^a^ Normal: 0–1000 followers; nano: 1000–10,000 followers; micro: 10,000–50,000 followers; mid-tier: 50,000–500,000 followers; macro: 500,000–1,000,000 followers. ^b^ An Instagram account for business enables brands to track their engagement and interactions and offer more analytic tools than a personal account. ^c^ Other business categories include business and utility services, grocery and convenience stores, lifestyle services, food and personal goods, professional services, publishers, non-profits and religious organizations, local events, auto dealers, transportation and accommodation services, home goods stores, content and apps, government agencies, and home and auto. ^d^ An Instagram verified account means Instagram has confirmed that an account is the authentic presence of the public figure, celebrity or global brand it represents. ^e^ Other languages that are over 1% include Spanish, Estonian, German, Catalan, French, Dutch, Lithuanian, and Korean.

**Table 2:** Overall network metrics.

Graph Metric	Whole Network	Largest Component
Nodes	4526	532
Isolated nodes	3714	0
Directed edges	1082	829
Connected components	98	1
Graph density	0.004	0.006
Average degree	1.333	1.558
Average clustering coefficient	0.171	0.181

**Table 3:** Top five accounts with in-degree, out-degree, betweenness, and closeness centrality, respectively.

Degree Centrality	No. of Posts	No. of Followers	Business Account	“iqos” in Username
**In-degree**				
0.02466	26	41,109	Yes	Yes
0.01849	72	11,470	Yes	Yes
0.01233	11	623	No	No
0.01233	49	1157	Yes	Yes
0.01110	4	1300	Yes	No
**Out-degree**				
0.14920	8	224	Yes	Yes
0.06782	3	257	Yes	Yes
0.03576	2	583	No	No
0.02959	16	446	Yes	No
0.02959	1	3658	No	No
**Betweenness**				
0.00799	49	1157	Yes	Yes
0.00437	3	257	Yes	Yes
0.00330	1	3658	No	No
0.00266	19	1546	Yes	Yes
0.00260	1	140,628	No	No
**Closeness**				
0.03094	72	11,470	Yes	Yes
0.02984	26	41,109	Yes	Yes
0.02436	49	1157	Yes	Yes
0.02270	11	623	No	No
0.02234	5	365	Yes	Yes

**Table 4:** Characteristics and example post of central nodes.

Category	Characteristics	Example Post
IQOS-associated (N = 6)	IQOS Instagram account representing a country/region; IQOS retailer and reseller stores; IQOS case/skin service provider	* 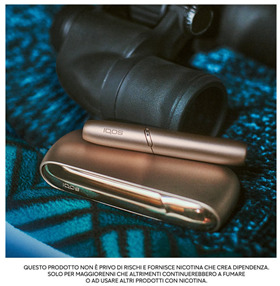 *
Tobacco-associated (N = 5)	IQOS or other general tobacco products retailer, tobacco product service provider	* 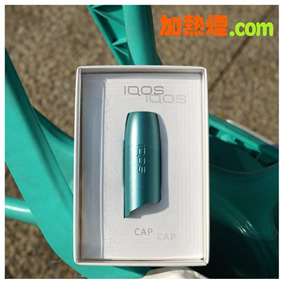 *
Others ^a^ (N = 2)	Celebrity and social media influencers	* 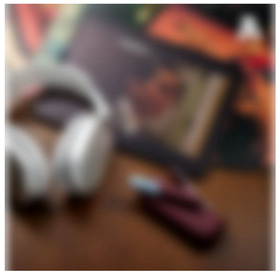 *

^a^ The example for “Others” was blurred due to privacy concerns.

## Data Availability

Data sharing not applicable.

## References

[1] Caputi T.L. (2017). Industry watch: Heat-not-burn tobacco products are about to reach their boiling point. Tob. Control.

[2] Tabuchi T., Gallus S., Shinozaki T., Nakaya T., Kunugita N., Colwell B. (2018). Heat-not-burn tobacco product use in Japan: Its prevalence, predictors and perceived symptoms from exposure to secondhand heat-not-burn tobacco aerosol. Tob. Control.

[3] Grand View Research (2020). Heated Tobacco Products Market Size, Share & Trends Analysis Report by Component (Capsules, Heating Module, Sticks, Vaporizers), by Distribution Channel, by Region, and Segment Forecasts, 2020–2027. https://www.grandviewresearch.com/industry-analysis/heat-not-burn-market.

[4] Glantz S.A. (2018). Heated tobacco products: The example of IQOS. Tob. Control.

[5] Bialous S.A., Glantz S.A. (2018). Heated tobacco products: Another tobacco industry global strategy to slow progress in tobacco control. Tob. Control.

[6] Chun L., Moazed F., Matthay M., Calfee C., Gotts J. (2018). Possible hepatotoxicity of IQOS. Tob. Control.

[7] Moazed F., Chun L., Matthay M.A., Calfee C.S., Gotts J. (2018). Assessment of industry data on pulmonary and immunosuppressive effects of IQOS. Tob. Control.

[8] Nabavizadeh P., Liu J., Havel C.M., Ibrahim S., Derakhshandeh R., Iii P.J., Springer M.L. (2018). Vascular endothelial function is impaired by aerosol from a single IQOS HeatStick to the same extent as by cigarette smoke. Tob. Control.

[9] St.Helen G., Iii P.J., Nardone N., Benowitz N.L. (2018). IQOS: Examination of Philip Morris International’s claim of reduced exposure. Tob. Control.

[10] Paumgartten F. (2018). Heat-not-burn and electronic cigarettes: Truths and untruths about harm reduction. Rev. Assoc. Med. Bras. (1992).

[11] Farsalinos K.E., Yannovits N., Sarri T., Voudris V., Poulas K. (2018). Nicotine delivery to the aerosol of a heat-not-burn tobacco product: Comparison with a tobacco cigarette and e-cigarettes. Nicotine Tob. Res..

[12] Kim M., Watkins S.L., Koester K.A., Mock J., Kim H.C., Olson S., Harvanko A.T., Ling P.M. (2020). Unboxed: US young adult tobacco users’ responses to a new heated tobacco product. Int. J. Environ. Res. Public Health.

[13] O’Brien E.K., Hoffman L., Navarro M.A., Ganz O. (2020). Social media use by leading US e-cigarette, cigarette, smokeless tobacco, cigar and hookah brands. Tob. Control.

[14] Freeman B., Chapman S. (2010). British American Tobacco on Facebook: Undermining article 13 of the global World Health Organization Framework Convention on Tobacco Control. Tob. Control.

[15] Huang J., Kornfield R., Szczypka G., Emery S.L. (2014). A cross-sectional examination of marketing of electronic cigarettes on Twitter. Tob. Control.

[16] Emery S.L., Vera L., Huang J., Szczypka G. (2014). Wanna know about vaping? Patterns of message exposure, seeking and sharing information about e-cigarettes across media platforms. Tob. Control.

[17] Payne J.D., Orellana-Barrios M., Medrano-Juarez R., Buscemi D., Nugent K. (2016). Electronic Cigarettes in the Media. Bayl. Univ. Med. Cent. Proc..

[18] Paek H.-J., Kim S., Hove T., Huh J.Y. (2014). Reduced harm or another gateway to smoking? Source, message, and information characteristics of E-cigarette videos on YouTube. J. Health Commun..

[19] Czaplicki L., Kostygina G., Kim Y., Perks S.N., Szczypka G., Emery S.L., Vallone D., Hair E.C. (2020). Characterising JUUL-related posts on Instagram. Tob. Control.

[20] Barker J.O., Vassey J., Chen-Sankey J.C., Allem J.P., Cruz T.B., Unger J.B. (2021). Categorizing IQOS-Related Twitter Discussions. Int. J. Environ. Res. Public Health.

[21] The Campaign for Tobacco-Free Kids (2019). Over 125 Organizations Call on Social Media Companies to End all Tobacco Advertising, Including by Paid Influencers. https://www.tobaccofreekids.org/press-releases/2019_05_21_socialmedia_advertising.

[22] The Campaign for Tobacco-Free Kids (2019). New Facebook/Instagram Policy on Tobacco Marketing Must be Immediately Implemented and Strictly Enforced. https://www.tobaccofreekids.org/press-releases/2019_12_18_facebook_instagram_marketing_policy.

[23] Kreitzberg D.S., Murthy D., Loukas A., Pasch K.E. (2019). Heat-not-burn tobacco promotion on instagram. Addict. Behav..

[24] Hejlová D., Schneiderová S., Klabíková Rábová T., Kulhánek A. (2019). Analysis of Presumed IQOS Influencer Marketing on Instagram in the Czech Republic in 2018–2019. Adiktologie.

[25] Vassey J., Metayer C., Kennedy C.J., Whitehead T.P. (2020). # Vape: Measuring e-cigarette influence on Instagram with deep learning and text analysis. Front. Commun..

[26] (2021). Instaloader v4.8.2. https://instaloader.github.io/contributing.html.

[27] Nobles A.L., Leas E.C., Latkin C.A., Dredze M., Strathdee S.A., Ayers J.W. (2020). # HIV: Alignment of HIV-related visual content on instagram with public health priorities in the US. AIDS Behav..

[28] Langdetect v1.0.9. https://pypi.org/project/langdetect/.

[29] Instagram Get Your Business Started on Instagram. https://business.instagram.com/getting-started.

[30] Hawe P., Webster C., Shiell A. (2004). A glossary of terms for navigating the field of social network analysis. J. Epidemiol. Community Health.

[31] Bastian M., Heymann S., Jacomy M. Gephi: An open source software for exploring and manipulating networks. Proceedings of the International AAAI Conference on Weblogs and Social Media.

[32] Costello C.D. (2018). “Hello? Are You Still There?” The Impact of Social Media on Self-Disclosure and Reciprocity in Interpersonal Relationships: A Literature Review. Channels Where Discip. Meet.

[33] The Campaign for Tobacco-Free Kids (2019). IQOS Social Media Examples. https://www.tobaccofreekids.org/media/2019/iqos-marketing.

[34] Instagram Help Center Edit Your Business Information on Instagram. https://help.instagram.com/529483457260403.

[35] Instagram Business Categories: The Complete Guide. https://boosted.lightricks.com/instagram-business-categories-the-complete-guide/.

[36] Instagram Is Testing Geo-Restriction for Stories and Posts. https://thenextweb.com/news/instagram-is-testing-geo-restriction-for-stories-and-posts.

